# Hospital Context Determinants of Variability in Healthcare-Associated Infection Prevalence: Multi-Level Analysis

**DOI:** 10.3390/microorganisms12122522

**Published:** 2024-12-07

**Authors:** Rui Malheiro, André Amaral Gomes, Carlos Fernandes, Ana Fareleira, Ana Lebre, Dulce Pascoalinho, João Gonçalves-Pereira, José-Artur Paiva, Rita Sá-Machado

**Affiliations:** 1 EPIUnit—Instituto de Saúde Pública, Universidade do Porto, Rua das Taipas, n° 135, 4050-600 Porto, Portugal; rmcoelho@med.up.pt; 2 Departamento de Ciências da Saúde Pública e Forenses, e Educação Médica, Faculdade de Medicina, Universidade do Porto, 4200-319 Porto, Portugal; 3 Unidade de Saúde Pública São João, Unidade Local de São João, 4200-510 Porto, Portugal; 4 Serviço de Medicina Intensiva, Hospital CUF, 4100-180 Porto, Portugal; andreamaralgomes@gmail.com; 5 Departamento de Medicina, Faculdade de Medicina, Universidade do Porto, 4200-319 Porto, Portugal; 6 Grupo Infeção e Sepsis, 4150-375 Porto, Portugal; dulcepascoalinho@gmail.com (D.P.); joaogpster@gmail.com (J.G.-P.); 7 Unidade Local de Saúde Coimbra, 3004-561 Coimbra, Portugal; cfernandes@chuc.min-saude.pt; 8 Serviço de Cirurgia Geral, Unidade Local de Saúde São João, 4200-319 Porto, Portugal; ana.fareleira@ulssjoao.min-saude.pt; 9 Serviço de Doenças Infeciosas/Unidade Local do Programa de Prevenção e Controlo de Infeção e Resistência aos Antimicrobianos, Instituto Português de Oncologia do Porto Francisco Gentil, E. P. E., 4200-072 Porto, Portugal; analebre@dgs.min-saude.pt; 10 Direção-Geral de Saúde, 1000-123 Lisboa, Portugal; ritasamachado@dgs.min-saude.pt; 11 Serviço de Medicina Intensiva, Unidade Local de Saúde Litoral Alentejano, 7540-230 Santiago do Cacém, Portugal; 12 Serviço de Medicina Intensiva, Unidade Local de Saúde Estuário do Tejo, 2600-009 Vila Franca de Xira, Portugal; 13 Clínica Universitária de Medicina Intensiva, Faculdade de Medicina, Universidade de Lisboa, 1649-004 Lisboa, Portugal; 14 Serviço de Medicina Intensiva, Unidade Local de Saúde São João, 4200-319 Porto, Portugal

**Keywords:** context, healthcare-associated infections, hospital departments, multi-level, prevalence

## Abstract

Healthcare-associated infections (HAIs) represent a major challenge in patient safety that affects services disproportionally. This paper aimed to assess how the HAI prevalence varies between hospital services and what contextual characteristics may explain such variance. A cross-sectional study was conducted on adult patients in Portuguese hospitals, using data from the European point prevalence survey of HAI prevalence. The study variables included patient, structural, and process variables, tested as risk factors, with patients clustered in hospitals. Variables with a *p*-value ≤ 0.2 in univariate analyses were retested in a multivariable model. A total of 18,261 patients from 119 hospitals were included: 736 from 56 intensive care units (ICUs), 3160 from 72 surgical departments, and 8081 from 90 medical departments. The HAI prevalence was 7.9%, 5.9%, and 1.7%, respectively. In ICUs, only the number of devices was associated with the HAI prevalence. In surgical departments, age, comorbidities, being a specialized hospital, and a higher ratio of infection prevention and control (IPC) personnel were associated with higher SSI. The safety climate was associated with lower SSI. In medical departments, age and devices were positively associated, whereas a larger ratio of IPC nurses was negatively associated. These results may help implement targeted interventions to achieve optimal results in each department.

## 1. Introduction

Healthcare-associated infections (HAIs) represent one of the major challenges in patient safety and are associated with increased mortality, length of stay, morbidity, and health expenditure. In Europe alone, they are estimated to represent almost 2.5 million disability-adjusted life years [1,2]. In the last point prevalence survey (PPS) of HAIs and antimicrobial use in European acute care hospitals by the European Centre for Disease Prevention and Control (ECDC), in 2023, Portugal had an HAI prevalence of 9.9%, after validation. The result was above the ECDC projection, when adjusted for patient case-mix and hospital characteristics. However, the prevalence was higher when considering the main types of HAIs: 17.5% for surgical site infections (SSIs), 23.2% for urinary tract infections, 17.5% for pneumonia, and 11.4% for bloodstream infections, of which 1.3% were related to central venous catheters [3]. 

The magnitude of the problem had already led the Directorate General of Health to establish, back in 2013, HAIs as a priority problem in the country, by creating a national program of infection prevention and control [4]. In that regard, Portugal is part of the Healthcare-Associated Infection Surveillance Network of the ECDC, which harmonizes the methodology for the surveillance of HAIs in order to improve outcomes and compare the effectiveness of key preventive measures [5] and is currently implementing an improvement project in 21 public hospitals to decrease the incidence of the four main types of HAIs by 50% [6].

Extensive research has demonstrated how compliance with hand hygiene [7] and the implementation of preventive strategies in a bundle approach [8,9,10,11] may help decrease the risk of these infections. However, the role of structural variables—both infrastructure and human resources—and process variables, namely those related to surveillance and infection prevention programs, remains to be established and has seldom been researched. Published systematic reviews suggest that higher nurse staffing and the establishment of surveillance for over 5 years may be associated with a decreased incidence of hospital-acquired pneumonia in intensive care units (ICUs) and of SSIs after colorectal surgery, respectively [12,13,14]. More research is needed to fully comprehend which contextual variables influence patient outcomes and how.

Better outcomes require improved processes and adequate resources [15]. Therefore, understanding the relationship between the context and HAIs is essential to optimize patient safety in the hospital setting. At the same, HAIs are heterogeneous and affect services disproportionally, not only in terms of their overall incidence but also in terms of the relative frequency of specific HAIs [3]. Thus, it is relevant to understand how structure and process indicators relate to the different HAIs in each major department, to implement maximally effective targeted interventions. One size may not fit all.

As such, this study aimed to assess whether HAI prevalence varies between hospitals, ICUs, and surgical and medical departments and whether such variance may be explained by hospital characteristics, resources, or implemented multimodal strategies.

## 2. Methods

### 2.1. Study Protocol

A cross-sectional study was conducted in Portuguese hospitals participating in the PPS of the ECDC, following their protocol [16], and under the supervision of the Portuguese Program for Infection and Antimicrobial Resistance Prevention of the Directorate General of Health. Healthcare professionals collected data from patient medical records and nurses’ notes, using the ECDC’s forms, with closed fields. The protocol was available in Portuguese to address any potential doubt in the retrieval of data, to ensure reproducibility and concordance of information, which were then registered using the HelicsWin software application, version 4.7.4, provided by the ECDC. All data collection in each ward was performed on one single day. Data from all hospitals were collected between 15 May and 19 May 2023, except for one hospital, which collected data on 30 May 2023. Patients younger than 18 years of age were excluded from this study. One hospital was excluded, as it did not provide data on structural and process variables. This study was not registered in a public database.

### 2.2. Study Variables

Data were collected at hospital and patient levels. Data at the individual patient level included the patient’s age, sex, McCabe score [17], which assesses the severity of patients’ comorbidities (categorized as ultimately fatal, rapidly fatal, non-fatal, or unknown), and device use (central vascular catheter, intubation, and/or urinary catheter).

The structural variables included the total number of acute beds, categorized as <250 beds, 250–500 beds, and >500 beds, total number of isolation rooms in the hospital, hospital type (primary, secondary, specialized, and tertiary), and location by health administrative region, divided into Norte, Lisboa e Vale do Tejo (LVT), Centro, and other, the latter encompassing the hospitals from Algarve, Alentejo, Madeira, and Azores. The ratio of doctors and nurses working on a full-time equivalent (FTE) basis in infection control per hospital bed and the number of professionals working on antimicrobial stewardship per hospital bed were also included, with 1 FTE being equal to a full-time worker. The existence of an infection prevention and control plan and report approved by the hospital administration, as a binary response, was also included.

The process variables included the use of masks, either in routine care or universally; hospital participation in surveillance networks for SSIs, HAIs in ICUs, *Clostridioides difficile* infection, antimicrobial consumption, and antimicrobial resistance; and availability of clinical tests or screening tests on weekends, to estimate microbiological performance during weekends.

Furthermore, data on multimodal strategy use for the implementation of infection prevention and control (IPC) interventions were collected, following the World Health Organization’s core component 5 of infection prevention [18]. Having an IPC multidisciplinary team, a regular link to develop multimodal strategies with colleagues, and the use of bundles or checklists were registered as binary responses (yes or no). System change, education and training, monitoring and feedback, communication and reminders, and safety climate and culture change were each assessed with a three-item Likert scale regarding their inclusion in multimodal strategies (not included, partially included, and fully included). The exact definitions are available in the point prevalent survey protocol and in Supplementary Table S1 [16].

### 2.3. Statistical Analysis

Each observation corresponded to one patient. Descriptive data are reported as the absolute and relative frequency and medians with the interquartile range, where applicable. The analysis considered patients clustered in hospitals, in a two-level hierarchical data structure. The variance of the outcome was given by τ^2^. An initial model was built for the entire study population, using a multi-level logistic regression, by fitting fully unconditional random-effects models with random intercepts at the hospital level. This model was used to assess the variance in HAIs between hospitals. Only catheter-line-associated bloodstream infections (CLABSIs), SSIs, catheter-associated urinary infections (CAUTIs), and intubation-associated pneumonia (IAP) were considered in the outcome since these represent not only the more prevalent and relevant infections but also those which are under local and national surveillance, following the HAI-Net protocol [5]. The codes from the ECDC’s PPS used to define these infections are available in Supplementary Table S2, and the flowchart of the inclusion criteria for HAIs is available in Supplementary Figure S1. The null model was used to estimate the intraclass cluster coefficient (ICC), which provides the proportion of total observed individual variation in the outcome that may be attributable to between-hospital variation. It is estimated as τ^2^/(τ^2^ + (π^2^/3)), where π refers to the mathematical constant, approximately equal to 3.14159. The same model was used to estimate the median odds ratio (MOR), defined as the median value of the odds ratio (OR) between the hospital at highest risk and the hospital at lowest risk of infection, when randomly selecting two patients with the same covariates from different hospitals.

The study variables were tested in a univariate multi-level logistic regression as risk factors for infection. Variables with a *p*-value ≤ 0.2 in univariate analysis were tested in a multivariable model [19]. A two-sided significance level of 0.05 was considered. Collinearity was assessed in the multivariable model using the generalized variance inflation factor (GVIF). To make the GVIF comparable across dimensions, GVIF^(1/(2×Df))^, where Df represents the number of coefficients in the subset, was used [20]. A value of 5 or above was interpreted as the presence of collinearity, and any variable meeting the threshold was dropped. The contextual effect of each included variable was given by its β-coefficient and respective *p*-value.

The analysis was repeated for three different subgroups: ICUs, surgical departments, and medical departments. For the analysis of ICUs, stroke and coronary units were not included since patients in these units have low clinical severity which would hamper meaningful associations. The outcome considered the same infections as for the entire population. In surgical departments, the outcome considered was SSIs alone, as these are the only HAIs under surveillance in these wards. Hence, admitted patients without previous surgery were excluded in this sub-analysis. In medical departments, the outcome was CAUTI, which is the most prevalent infection in these wards and the one under surveillance in this context.

The analysis was performed using R, version 4.4.0, using the ‘merTools’, ‘lme4’, and ‘car’ packages.

## 3. Results

**Figure 1:**
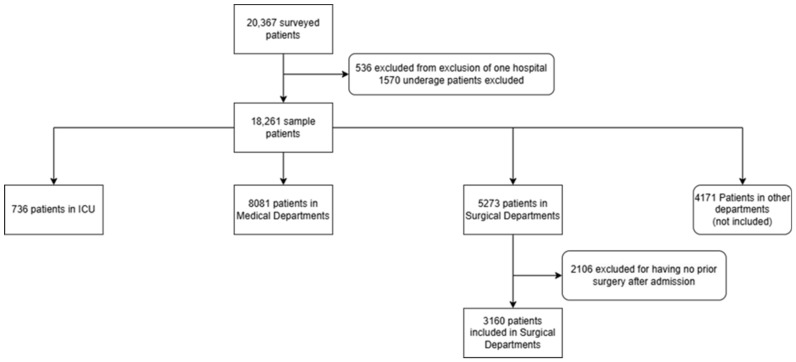
Flowchart of patient inclusion, by service or department.

A total of 18,261 patients from 119 hospitals were included in the analysis, of which 736 were in 56 ICUs, 3160 were in 72 surgical departments, and 8081 were in 90 medical departments (Figure 1). The overall prevalence of any HAI under surveillance (SSI, CLABSI, IAP, and CAUTI) was 3.5%. The baseline characteristics of the population, both at the individual and hospital levels, are presented in Table 1, for the entire population and per department. Most hospitals had fewer than 250 beds, although patients were almost evenly distributed across differently sized hospitals. The HAI prevalence was 7.9% in ICUs, whereas the SSI prevalence was 5.9% in surgical departments, and the CAUTI prevalence was 1.7% in medical departments.

**Figure 2:**
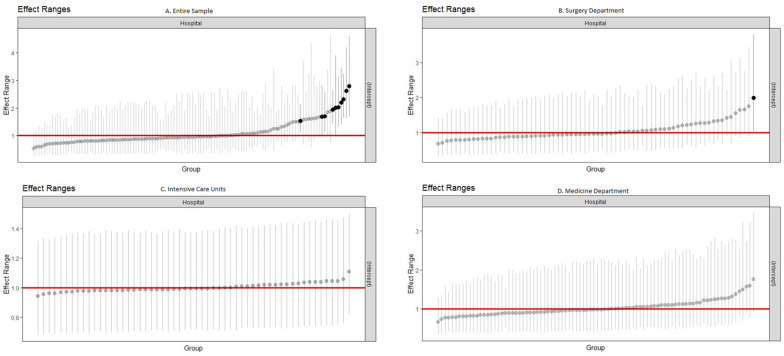
Simulated random effects of the clustering of hospitals on healthcare-associated infection prevalence. Note: Effect is presented as the odds ratio between each hospital and the overall average. In the overall sample, catheter-associated bloodstream infection (CLABSI), intubation-associated pneumonia (PAI), surgical site infection (SSI), and catheter-associated urinary tract infection (CAUTI) are used as the outcome. Intensive care units consider CLABSI, PAI, and CAUTI. Surgical departments consider SSI as the outcome, and medical departments consider CAUTI.

The estimated variance of the random effects in the null model was 0.2579, corresponding to an ICC of 0.07 and an MOR of 1.62. The surgical and medical departments had a variance of 0.1875 and 0.1796 and an MOR of 1.51 and 1.50, respectively, both with an ICC of 0.05. The variance in ICUs was markedly lower, −0.0299, corresponding to an ICC of 0.01 and an MOR of 1.18. The simulated random effects are plotted in Figure 2. The effect is presented as the odds ratio between each hospital and the overall average.

The univariate and multivariable analyses are presented in Table 2, overall and per department. A total of 15 variables were included in the multivariable multi-level logistic regression model for the entire hospital. Age and the number of devices were identified as individual-level variables associated with a higher risk of infection, whereas being in the fourth quartile in the number of isolation rooms, being in the fourth quartile in the ratio of infection prevention doctors to number of acute beds, being in the second and third quartiles in the ratio of infection prevention nurses to number of acute beds, and the use of masks, both universally and in care, were significantly associated with higher infection at the hospital level. Having screening tests on one day at the weekend, as well as having additional methods or initiatives to improve team communication across units and disciplines, when compared to the use of reminders, posters, or advocacy/awareness-raising tools, was significantly associated with lower infection.

In ICUs, only the number of devices inserted in each patient was associated with the HAI prevalence. No contextual variable included in the multivariable analysis met the threshold.

In surgical departments, older age, ultimately fatal comorbidities assessed by the McCabe score, being in a specialized hospital, and having more infection prevention doctors and nurses per acute bed were significantly associated with SSI. Full investment in the safety climate of the department, with the empowerment of teams and individuals, when compared to no inclusion of this element in a multimodal strategy for infection prevention and control was associated with lower SSI.

In medical departments, age and the number of devices were significantly associated with CAUTI, and a larger ratio of nurses in infection prevention per hospital acute bed (fourth quartile vs. first quartile) was associated with decreased CAUTI. In all models, no variable had to be removed due to collinearity, as GVIF^(1/(2*Df))^ was always below 5 for every included variable (Supplementary Table S3).

## 4. Discussion

This study found that the structural and process variables associated with HAIs may be different depending on the services or department considered within hospitals and on the specific types of HAIs. In ICUs, the variance is much lower, and the number of devices per patient is the only variable associated with HAI prevalence. In surgical and medical departments, however, several contextual variables appear to influence the HAI prevalence.

The apparent immunity of ICUs to contextual variables may be because ICUs are more similar than surgical or medical departments, which encompass a variety of different services whose distribution is affected by the hospital size and type. At the same time, the workflow in ICUs relies more heavily on detailed protocols and procedures than that in their counterparts, which may translate to a more systematic and standardized approach to patient care, hence, with lesser variability between settings. Nonetheless, variability is often assessed considering costs as a proxy for patient care, and quantification is not always easy [21,22]. Finally, it is worth considering that the effect of devices may be so significant, when their prevalence is highest, as is the case of ICUs, that it may overshadow other smaller potential determinants. These hypotheses require further research, directed to the questions at hand, since most research in the field has disregarded the potential role of contextual effects [23,24,25].

In surgical departments, the empowerment of teams and individuals as owners and protagonists of quality improvement interventions and the existence of a safety climate and psychological comfort were found to have a negative association with the HAI prevalence. The visible support and commitment of managers and leaders in strengthening a culture of patient safety was not significantly associated. These results emphasize the role of quality improvement interventions in the battle against HAIs. Portugal implemented such a program, between 2015 and 2018, named *Stop Infeção Hospitalar*! It aimed to reduce the incidence rate of the four typologies of HAIs researched here—CLABSI, CAUTI, IAP, and SSI—in 12 hospitals [26]. Portugal is currently developing a similar program, called *Stop Infeção Hospitalar*-2.0, involving 22 acute care hospitals and aiming at a 50% percent reduction, in three years, in the incidence of the same HAIs [6]. This suggests that, at least in surgical departments, the success of the project lies both in the quality of the proposed bundles and the compliance with them and in the philosophy of local teams and professionals, their empowerment, psychological safety, and cooperation and participation in the endeavor. This has been also emphasized in other studies [27]. Safety culture change was marginally insignificant in the entire population and almost similar to chance in medical departments, raising the question of whether the effect of this strategy may be different in different services and departments, depending on the professionals and department cultures.

When considering all services from the included hospitals, an investment in active communication beyond the use of common awareness-raising tools was found to be significantly associated with a smaller prevalence of HAIs. Even though the implementation of the IPC assessment framework has recently been shown to be associated with a decreased prevalence of HAIs [28], to the best of our knowledge, no study has underlined the individual role of communication as a main driver for better health results. However, communication strategies have been found to be associated with improved healthcare delivery results in different contexts [29,30], and our finding supports a renewed investment in this research field.

The number of isolation rooms was also found to be positively associated with a lower HAI prevalence in the entire sample, when adjusted for hospital bed size and type. This result was not found in the sub-analyses per service and department. However, the variable relates to the number of isolation rooms in the hospital, not per service, and therefore, it may have no practical meaning in the sub-analyses performed. Likewise, the availability of screening tests on weekends was found to have a negative association with the HAI prevalence at the entire sample level only. Isolation rooms were found to reduce the rate of HAIs in pediatric ICUs and in the COVID-19 context [31,32], but published papers are lacking, suggesting these results should be interpreted with caution, warranting further research.

The positive association between the ratio of IPC nurses and doctors per acute bed and the prevalence of HAIs, which was found in the entire sample but also in surgical and medical departments, should not be necessarily interpreted as a causative association and it should not lead to the immediate conclusion that an investment in specialized personnel is deleterious to infection prevention efforts but could rather mean that an artificial increase in HAI rates may occur. This artificiality is well supported in the literature, which consistently found that better registry, active post-discharge case finding, and better audits are associated with reports of higher infection rates [33,34]. This effect was not found when the participation in surveillance networks was assessed, even in specific cases, as was the case of participation in the surveillance network of HAIs in ICUs and participation in the surveillance network of SSIs in surgical departments. However, the cross-sectional nature of this paper may fail to find the parabolic effect of surveillance on HAIs, which tends to artificially increase rates and then reduce them after the 5-year mark [12]. At the same time, the high ratio of IPC personnel may reflect a prior response to the higher prevalence of infection and represent a consequence of high HAI rates. Due to the transversal nature of the analysis, one may not clearly define the direction of the association found. Nevertheless, published research supports the notion that increased staff levels are protective in terms of medical complications [14].

Our research also found that, although univariate analyses suggest that hospital size may have a role, albeit indirect, in HAI rates, such an effect was unapparent after adjustment to other variables. Even though the paper by Zingg et al. did find a significant association, they compared larger hospitals to smaller hospitals, with a cut-off at 650 beds, which is hardly meaningful in many national settings [19].

To the best of our knowledge, this is the first paper to consider that different departments may benefit from different IPC strategies and have different determinants of their success. Few papers have used the ECDC’s PPS to assess contextual factors in the HAI prevalence in entire hospitals [19,35,36]. Barbadoro et al. and Zingg et al. both used a multi-level logistic regression approach, yet the former performed bivariate analysis only. The latter found that larger, tertiary care hospitals had a positive association with the HAI prevalence, as discussed, and that private for-profit hospitals had a negative association [16,29]. Palaiopanos et al. followed a different approach and used a multivariable linear regression analysis of hospital factors associated with HAIs [28]. Bed occupancy was the only variable with a significant—and positive—association. The large sample of our study, obtained by the participation of virtually every hospital in the country, together with the inclusion of multimodal strategies’ implementation, strengthens the internal validity of our findings. The inclusion of HAIs that are currently under surveillance in each local setting makes our findings more relatable to everyday professionals in infection prevention and control, improving the practicality of interventions that address some of the highlighted issues presented.

This study has some limitations. As a cross-sectional study, it disallows researchers from drawing causality from any findings, and the absence of a significant association does not mean that the variable is not associated with infection. This paper addresses variance between institutions, and hence, every time hospitals share an intervention—having a multidisciplinary team—or fail systematically to do so, the variable will fail to significantly explain any variation, regardless of its real-world potential association with HAI rates. Also, some variables have inherent limitations, as is the case of the implementation of bundles or checklists, which are considered together despite being two diverse things. Besides the limitation of being unable to consider bundles and checklists separately in the analysis, it is also possible that the interpretation of multimodal strategies’ inclusion may have not been consistent throughout the hospitals, although random variation is not expected to affect the validity of our findings. Finally, the high proportion of unknown values in multimodal strategies’ implementation may have hindered the potential conclusions regarding their effect since missing values may have biased the comparison.

## 5. Conclusions

Different hospital departments seem to have different infection prevention and control needs and potentially benefit from different ICP interventions and strategies. The results of this paper may help implement targeted interventions that may achieve optimal results in each department, considering the specificities of local realities.

## Figures and Tables

**Table 1:** Baseline characteristics of the population.

Hospital Variables	All Hospitals	ICU Department	Surgical Department	Medical Department
**No. hospitals**	119	56	72	90
**No. patients**	18,261	736	3160	8081
**No. patients with HAI (prevalence)**	638 (3.5)	58 (7.9)	187 (5.9)	138 (1.7)
**Age (median [IQR])**	71 (56–82)	67 (55–76)	68 (56–78)	76 (64–85)
**Male sex**	9182 (50.3)	455 (61.2)	1653 (52.3)	4184 (51.8)
**McCabe score**				
Ultimately fatal	4068 (22.3)	148 (19.9)	524 (16.6)	2437 (30.1)
Rapidly fatal	1033 (5.7)	72 (9.7)	67 (2.1)	648 (8.0)
Non-fatal	12,943 (70.9)	500 (67.2)	2531 (80.1)	4931 (61.0)
Unknown	204 (1.1)	16 (2.2)	38 (1.2)	65 (0.8)
**Device use**				
CVC	1529 (8.4)	461 (62.0)	285 (9.0)	506 (6.3)
Urinary catheter	4250 (23.3)	585 (78.6)	726 (23.0)	1906 (23.6)
Intubation	461 (2.5)	277 (37.2)	50 (1.6)	84 (1.0)
Has any device	5045 (27.6)	608 (81.7)	912 (29.2)	2309 (28.6)
**Hospital bed size**				
0–250	6109 (28.0)	130 (17.5)	703 (22.2)	2273 (28.1)
251–500	6264 (34.3)	290 (39.0)	1116 (35.3)	2537 (31.4)
>500	6888 (37.7)	316 (52.5)	1341 (42.4)	3271 (40.5)
**Hospital type**				
Primary	1366 (7.5)	34 (4.6)	127 (4.0)	727 (9.0)
Secondary	7248 (39.7)	233 (31.3)	930 (29.4)	3585 (44.4)
Specialized	840 (4.6)	19 (2.6)	208 (6.6)	195 (2.4)
Tertiary	8807 (48.2)	450 (60.5)	1895 (60.0)	3574 (44.2)
**Hospital location**				
Norte	6146 (33.7)	311 (41.8)	1221 (38.6)	2319 (828.7)
Centro	3488 (19.1)	88 (11.8)	633 (20.0)	1461(18.1)
Lisbon	6083 (33.3)	261 (35.1)	877 (27.8)	3056 (37.8)
Other	2544 (13.9)	76 (10.2)	429 (13.6)	1245 (15.4)
**No. hospital isolation rooms**	5 (1–10)	8 (2–14)	6 (1–8)	5 (1–8)
**Clinical tests on weekend**				
Both days	10,140 (55.5)	463 (62.2)	314 (9.9)	4390 (54.3)
One day only	2329 (12.8)	48 (6.5)	1851 (58.6)	1394 (17.2)
**Screening tests on weekend**				
Both days	10,567 (57.9)	470 (63.2)	492 (15.6)	4610 (57.0)
One day only	3079 (16.9)	77 (10.3)	1927 (61.0)	1604 (19.9)
IPC doctors-to-acute beds ratio (median [IQR])	0.1 (0.0–0.2)	0.1 (0.0–0.3)	0.1 (0.0–0.3)	0.1 (0.0–0.2)
IPC nurses-to-acute beds ratio (median [IQR])	0.5 (0.4–0.7)	0.5 (0.4–0.7)	0.5 (0.4–0.8)	0.5 (0.4–0.7)
AMS consultants-to-acute beds ratio (median [IQR])	0.1 (0.0–0.1)	0.1 (0.0–0.1)	0.1 (0.0–0.1)	0.0 (0.0–0.1)
CEO-approved IPC plan	14,890 (81.5)	630 (84.7)	2572 (81.4)	6560 (81.2)
CEO-approved IPC report	15,669 (85.8)	670 (90.1)	2781 (88.0)	6855 (84.8)
**Universal masking**				
Care	7833 (42.9)		1471 (46.6)	3169 (39.2)
Always	412 (2.3)		81 (2.6)	126 (1.6)
**Participation in surveillance networks**				
Surgical site infection	8597 (47.1)	-	1604 (50.8)	-
HAI in intensive care units	9171 (50.2)	418 (56.2)	-	-
*Clostridium difficile*	3715 (20.3)	123 (16.5)	495 (15.7)	1804 (22.3)
Antimicrobial resistance	10,900 (59.7)	453 (60.9)	1991 (63.0)	4839 (59.9)
Antimicrobial consumption	11,070 (60.6)	478 (64.2)	2070 (65.5)	4987 (61.7)
**Multimodal strategy use**				
**System change**				
Element not included	104 (0.6)	4 (0.5)	0 (0.0)	41 (0.5)
L1	3891 (21.3)	143 (19.4)	584 (18.5)	1906 (23.6)
L2	9786 (53.6)	445 (60.5)	1931 (61.1)	4010 (49.6)
Unknown	4480 (24.5)	144 (19.6)	645 (20.4)	2124 (26.3)
**Education and training**				
L1	2185 (12.0)	57 (7.7)	310 (9.8)	867 (10.7)
L2	11,596 (63.5)	535 (72.7)	2205 (69.8)	5090 (63.0)
Unknown	4480 (24.5)	144 (19.6)	645 (20.4)	2124 (26.3)
**Monitoring and feedback**				
Element not included	40 (0.2)	5 (0.7)	0 (0.0)	2 (0.02)
L1	3194 (17.5)	175 (23.8)	557 (17.6)	1426 (17.6)
L2	10,547 (57.8)	412 (55.9)	1958 (61.9)	4529 (56.0)
Unknown	4480 (24.5)	144 (19.6)	645 (20.4)	2124 (26.3)
**Communications and reminders**				
L1	8267 (45.3)	365 (49.6)	1554 (49.2)	3504 (43.4)
L2	5514 (30.2)	227 (30.8)	961 (30.4)	2453 (30.4)
Unknown	4480 (24.5)	144 (19.6)	645 (20.4)	2124 (26.3)
**Safety climate and culture change**				
Element not included	2702 (14.8)	80 (10.9)	413 (13.1)	1336 (16.5)
L1	5322 (29.1)	222 (30.2)	899 (28.4)	2442 (30.2)
L2	5685 (31.1)	290 (39.4)	1203 (38.1)	2125 (26.3)
Unknown	4552 (24.9)	144 (19.6)	645 (20.4)	2178 (26.6)
**Is a multidisciplinary team used to implement IPC multimodal strategies**				
Yes	12,620 (69.1)	535 (72.7)	2265 (71.8)	5350 (66.2)
No	1161 (6.4)	57 (7.7)	250 (7.9)	607 (7.5)
Unknown	4480 (24.5)	144 (19.6)	645 (20.4)	2124 (26.3)
**Link to developing multimodal strategy with colleagues**				
Yes	11,210 (61.4)	496 (67.4)	1981 (62.7)	4723 (58.4)
No	2571 (14.1)	96 (13.0)	534 (16.9)	1234 (15.3)
Unknown	4480 (24.5)	144 (19.6)	645 (20.4)	2124 (26.3)
**Bundles or checklists**				
Yes	11,596 (63.5)	587 (79.8)	2477 (78.4)	5762 (71.3)
No	2185 (12.0)	5 (0.68)	38 (1.2)	195 (2.4)
Unknown	4480 (24.5)	144 (19.6)	645 (20.4)	2124 (26.3)

Note: AMS, antimicrobial stewardship. CEO, Chief Executive Officer. HAI, healthcare-associated infection. IQR, interquartile range. IPC, infection prevention and control.

**Table 2:** Univariate and multivariable regression analysis of patient-level and hospital-level variables associated with healthcare-associated infections’ prevalence.

	All Hospitals				ICU				Surgery				Medicine			
	Univariate		Multivariable		Univariate		Multivariable		Univariate		Multivariable		Univariate		Multivariable	
Patient Variable	β	*p*-Value	β	*p*-Value	β	*p*-Value	β	*p*-Value	β	*p*-Value	β	*p*-Value	β	*p*-Value	β	*p*-Value
**Age**	0.01	**<0.001**	0.01	**<0.001**	−0.01	0.573	-	-	0.00	0.393	−6.32	**<0.001**	0.04	**<0.001**	0.04	**<0.001**
**Male sex**	0.20	**0.013**	0.12	0.204	0.45	0.146	0.39	0.225	0.45	**0.004**	0.31	0.07900	−0.19	0.274	-	-
**McCabe score**																
Non-fatal	ref		ref		ref		ref		ref		ref		ref		ref	
Rapidly fatal	0.25	0.138	−0.24	0.227	−0.04	0.940	-	-	0.94	0.0180	0.64	0.1600	−0.12	0.73	−0.30	0.445
Ultimately fatal	0.45	**<0.001**	0.15	0.163	0.30	0.381	-	-	0.91	**<0.001**	0.76	**<0.001**	0.31	0.09	−0.12	0.618
**Device no.**	0.92	**<0.001**	0.91	**<0.001**	1.06	**<0.001**	0.11	**<0.001**	0.28	**0.025**	0.06	0.6970	1.14	**<0.001**	1.49	**<0.001**
**Hospital variable**	**β**	***p*-value**	**β**	***p*-value**	**β**	***p*-value**	**β**	***p*-value**	**β**	***p*-value**	β	***p*-value**	**β**	***p*-value**	**β**	***p*-value**
**Hospital bed size**																
0–250	ref		ref		ref		ref		ref		ref		ref			
250–500	0.52	**0.001**	0.02	0.936	−0.46	0.259	-	-	0.38	0.150	−0.44	0.301	0.09	0.711	-	-
>500	0.67	**<0.001**	0.45	0.257	0.09	0.810	-	-	0.68	**0.013**	−0.73	0.342	−0.09	0.704	**-**	-
**Hospital type**																
Primary	ref		Ref		Ref		ref		Ref		Ref		Ref			
Secondary	0.20	0.416	−0.12	0.666	0.29	0.706	-	-	−0.25	0.571	−0.19	0.823	−0.09	0.794	-	-
Specialized	0.49	0.156	−0.89	0.076	−0.11	0.928	-	-	0.84	**0.080**	2.17	**0.029**	0.31	0.599	**-**	-
Tertiary	0.32	0.202	−0.02	0.949	0.28	0.708	-	-	0.11	0.795	0.84	0.309	−0.23	0.494	**-**	-
**Hospital location**																
Lisbon	Ref		-	-	Ref		ref		Ref				Ref		ref	
Centro	0.02	0.918	-	-	0.82	0.060	0.03	0.963	−0.13	0.667	-	-	0.09	0.765	0.29	0.534
Norte	0.13	0.481	-	-	0.39	0.263	−0.42	0.517	0.07	0.763	-	-	−0.08	0.766	−0.65	0.156
Other	0.044	0.846	-	-	0.730	0.115	−0.40	0.526	−0.09	0.779	-	-	0.38	0.196	0.06	0.913
**No. hospital isolation rooms**																
1st quartile	ref		ref		ref		ref		ref		ref		ref			
2nd quartile	0.48	**0.006**	0.07	0.745	0.31	0.433	−0.23	0.684	0.64	**0.014**	−0.65	0.188	0.19	0.49	-	-
3rd quartile	0.43	**0.044**	−0.30	0.244	0.50	0.193	0.04	0.936	0.65	**0.029**	−0.36	0.531	−0.27	0.393	-	-
4th quartile	0.37	0.060	−0.62	**0.011**	−0.31	0.502	−0.06	0.913	0.39	0.152	−0.8	0.131	−0.14	0.637	-	-
**Clinical tests on weekends**																
One day only	−0.31	0.247	0.09	0.852	−0.68	0.380	-	-	−0.45	0.246	-	-	−0.25	0.455	0.27	0.808
Both days	−0.33	0.055	0.93	0.762	0.07	0.843	-	-	−0.23	0.316	-	-	−0.32	0.172	0.87	0.530
**Screening tests on weekends**																
One day only	−0.13	0.603	−0.78	**0.026**	−0.44	0.475	-	-	−0.29	0.410	-	-	−0.55	0.104	−0.56	0.468
Both days	−0.30	0.104	−0.93	0.220	0.09	0.800	-	-	−0.21	0.420	-	-	0.42	0.089	−1.55	0.243
**IPC doctors-to-acute beds ratio**																
1st quartile	Ref		Ref		ref		ref		ref		ref		ref		ref	
2nd quartile	−0.24	0.188	0.27	0.185	−0.93	**0.033**	−0.24	0.672	0.11	0.686	1.31	**0.006**	−0.57	0.0531	0.12	0.797
3rd quartile	0.08	0.673	−0.03	0.909	−0.59	0.132	−0.03	0.958	0.41	0.147	0.99	**0.048**	−0.14	0.6146	1.06	0.063
4th quartile	0.29	0.145	0.50	**0.031**	−0.06	0.862	0.75	0.293	0.33	0.256	0.80	0.089	−0.14	0.6206	0.99	0.121
**IPC nurses-to-acute beds ratio**																
1st quartile	Ref		Ref		ref				ref		ref		ref		ref	
2nd quartile	0.27	0.118	0.62	**0.028**	0.27	0.531	0.33	0.557	0.48	0.071	2.44	**<0.001**	−0.13	0.666	0.09	0.848
3rd quartile	0.69	**<0.001**	0.70	**0.007**	0.53	0.189	0.68	0.255	0.52	0.052	2.20	**0.004**	0.42	0.104	0.55	0.167
4th quartile	0.42	**0.044**	0.25	0.936	0.15	0.743	0.00	1.000	0.66	**0.025**	2.09	**0.005**	−0.32	0.298	−0.95	**0.037**
**AMS consultants-to-acute beds ratio**																
1st quartile	Ref		-	-	ref		ref		ref				ref		ref	
2nd quartile	−0.04	0.841	-	-	−0.29	0.456	-	-	−0.22	0.424	-	-	0.02	0.941	0.28	0.451
3rd quartile	0.11	0.616	-	-	−0.47	0.247	-	-	0.31	0.234	-	-	−0.46	0.129	−0.17	0.736
4th quartile	0.23	0.269	-	-	−0.13	0.724	-	-	0.28	0.302	-	-	0.01	0.978	−0.30	0.572
**CEO-approved IPC plan**	0.01	0.967	-	-	0.01	0.979	-	-	0.26	0.324	-	-	−0.50	**0.0294**	−0.70	0.206
**CEO-approved IPC report**	0.18	0.39	-	-	0.59	0.337	-	-	0.22	0.472	-	-	−0.242	0.382	-	-
**Participation in surveillance network**																
SSI	0.15	0.418	-	-	-	-	-	-	0.12	0.636	-	-	-	-	-	-
ICU	0.15	0.377	-	-	0.32	0.332	-	-	-	-	-	-	-	-	-	-
CDI	0.09	0.642	-	-	−0.45	0.318	-	-	0.30	0.221	-	-	0.29	0.287	-	-
AMR	0.34	0.055	0.21	0.538	−0.35	0.314	-	-	0.87	**0.003**	0.84	0.237	0.48	0.102	−0.04	0.957
AMC	0.34	**0.039**	0.49	0.242	−0.46	0.185	−0.89	0.126	0.62	**0.011**	0.29	0.701	0.59	**0.0354**	1.10	0.15
**Universal masking**																
Care	0.39	**0.010**	0.44	**0.007**	0.25	0.430	-	-	0.32	0.173	0.19	0.627	0.67	0.726	**-**	-
Always	0.73	**0.039**	1.34	**0.012**	0.38	0.728	-	-	0.80	0.119	1.03	0.401	0.78	0.248	-	-
**Multimodal strategies**	**β**	***p*-value**	**β**	***p*-value**	**β**	***p*-value**	**β**	***p*-value**	**β**	***p*-value**	β	***p*-value**	**β**	***p*-value**	**β**	***p*-value**
**System change**																
L2 vs. L1	0.02	0.941	-	-	0.09	0.813	-	-	0.19	0.537	-	-	−0.31	0.201	-	-
**Education and training**																
L2 vs. L1	−0.15	0.508	-	-	0.50	0.412	-	-	−0.10	0.769	-	-	−0.48	0.089	0.08	0.885
**Monitoring and feedback**																
L2 vs. L1	−0.06	0.795	-	-	−0.18	0.578	-	-	−0.31	0.295	-	-	−0.30	0.255	-	-
**Communication**																
L2 vs. L1	−0.37	**0.048**	−0.46	**0.006**	−0.34	0.293	-	-	−0.03	0.912	-	-	−0.24	0.33	-	-
**Safety culture change**																
L1	−0.35	0.154	0.52	0.065	−0.43	0.375	-	-	−0.31	0.385	−0.87	0.175	−0.61	**0.0211**	−0.06	0.922
L2	−0.28	0.255	0.59	0.091	0.11	0.807	-	-	−0.60	0.096	−1.59	**0.019**	−0.64	**0.0223**	−0.01	0.987
**Multidisciplinary team**	−0.38	0.337	-	-	−0.10	0.847	-	-	−0.32	0.492	-	-	−0.61	0.0879	−0.43	0.561
**Link to developing strategies with colleagues**	−0.52	**0.025**	−0.42	0.151	0.17	0.749	-	-	−0.57	0.052	0.89	0.168	−0.24	0.397	-	-
**Bundles or checklists**	0.33	0.530	-	-	0.24	0.526	-	-	0.81	0.464	-	-	0.24	0.731	-	-

Note: AMC, antimicrobial consumption. AMR, antimicrobial resistance. AMS, antimicrobial stewardship. CDI, Clostridium difficile infection. CEO, Chief Executive Officer. ICU, intensive care unit. IPC, infection prevention and control. SSI, surgical site infection. Statistically significant results are presented in bold.

## Data Availability

The original contributions presented in the study are included in the article/Supplementary Material, further inquiries can be directed to the corresponding author/s.

## Supplementary Materials

The following supporting information can be downloaded at https://www.mdpi.com/article/10.3390/microorganisms12122522/s1: Figure S1: flowchart of infections inclusion, overall and per department; Table S1: Description of multimodal strategies core components levels; Table S2: Codes and criteria for inclusion of registered infections as healthcare-associated infections; Table S3: Collinearity analyses for multivariable multi-level logistic regression models.
