# Supplementary material for: Hospital Context Determinants of Variability in Healthcare-Associated Infection Prevalence: Multi-Level Analysis
Source: Microorganisms. 2024 Dec 7;12(12):2522. doi: 10.3390/microorganisms12122522 (PMC11676765; doi:10.3390/microorganisms12122522)
Supplement: Supplementary file 1 [file microorganisms-12-02522-s001.zip › Supp Table S1.pdf]

Supplementary Table S1. Description of multimodal strategies core components levels

Component	Description
**System Change**	
L1	Interventions to ensure the necessary infrastructure and continuous availability of supplies are in place
L2	Interventions to ensure the necessary infrastructure and continuous availability of supplies are in place and addressing ergonomics and accessibility (e.g. best placement of central venous catheter set and tray)
**Education and training**	
L1	Written information and/or oral instruction and/or e-learning *only*
L2	Additional interactive training sessions (includes simulation and/or bedside training)
**Monitoring and Feedback**	
L1	Monitoring compliance with process or outcome indicators (e.g. audits of hand hygiene or catheter practices)
L2	Monitoring compliance and providing timely feedback of monitoring results to healthcare workers and key players
**Communications and reminders**	
L1	Reminders, posters, or other advocacy/awareness-raising tools to promote the intervention
L2	Additional methods/initiatives to improve team communication across units and disciplines (e.g., by establishing regular case conferences and feedback rounds)
**Safety climate and culture change**	
L1	Managers/leaders show visible support and act as champions and role models, promoting an adaptive approach and strengthening a culture that supports IPC, patient safety and quality
L2	Additionally, teams and individuals are empowered so that they perceive ownership of the intervention (e.g., by participatory feedback rounds)

Note: IPC, infection prevention and control.
