# Supplementary material for: Hospital Context Determinants of Variability in Healthcare-Associated Infection Prevalence: Multi-Level Analysis
Source: Microorganisms. 2024 Dec 7;12(12):2522. doi: 10.3390/microorganisms12122522 (PMC11676765; doi:10.3390/microorganisms12122522)
Supplement: Supplementary file 1 [file microorganisms-12-02522-s001.zip › Supp Table S2.pdf]

Supplementary Table S2. Codes and criteria for inclusion of registered infections as healthcare-associated infections

Infection Type	Infection Site<br>ECDC's PPS Code	Invasive Device
CAUTI	UTI-A<br>UTI-B	Y
CLABSI	CRI1-CVC<br>CRI2-CVC<br>CRI3-CVC	Y
PAI	PN1<br>PN2<br>PN3<br>PN4<br>PN5	Y
SSI	SSI-D<br>SSI-O<br>SSI-S	-

Note: CAUTI, cateter-associated urinary tract infection. CLABSI, catheter-line associated bloodstream infection. ECDC. European Center for Disease Prevention and Control. PAI, pneumonia associated with intubation. PPS, point prevalence survey. SSI, surgical site infection.
