# Supplementary material for: Hospital Context Determinants of Variability in Healthcare-Associated Infection Prevalence: Multi-Level Analysis
Source: Microorganisms. 2024 Dec 7;12(12):2522. doi: 10.3390/microorganisms12122522 (PMC11676765; doi:10.3390/microorganisms12122522)
Supplement: Supplementary file 1 [file microorganisms-12-02522-s001.zip › Supp Table S3.pdf]

Supplementary Table S3. Collinearity analyses for multivariable multi-level logistic regression models

**All Wards**	**GVIF**	**Df**	**GVIF^(1/(2*Df))**
Age	1.085757	1	1.041997
Gender	1.052603	3	1.008581
McCabe Score	1.173775	3	1.027064
Device number	1.084364	1	1.041328
Hospital size	36.845694	2	2.46375
Hospital Type	39.625411	3	1.846413
Number of Isolation rooms	27.135452	3	1.733496
Clinical Tests on Weekends	133.506031	2	3.399188
Screening Tests on Weekends	160.86419	2	3.561352
IPC-Doctors-to-bed ratio	28.322508	3	1.74591
IPC-Nurses-to-bed ratio	41.173579	3	1.858246
Surveillance of AMR	23.838241	2	2.209625
Surveillance of AMC	30.355672	2	2.347253
Universal Masking	7.00703	2	1.626985
Communication as MM Strategy	2.963947	1	1.721612
Safety Culture Change	24.74415	2	2.230325
Link with colleagues	7.285394	1	2.699147
**Medical Wards**			
Age	1.105536	1	1.051445
McCabe score	1.234969	3	1.0358
Device number	1.086138	1	1.042179
Hospital Location	27.905129	3	1.741596
Clinical Tests on Weekends	112.186439	2	3.254506
Screening Tests on Weekends	90.207802	2	3.081847
IPC-Doctors-to-bed ratio	31.767458	3	1.779633
IPC-Nurses-to-bed ratio	13.913117	3	1.550853
Stewardship consultants ratio	18.583482	3	1.627501
CEO Approved IPC Plan	6.286935	1	2.507376
Surveillance of AMR	17.914596	2	2.05732
Surveillance of AMC	20.765168	2	2.134685
Safety Culture Change	16.474472	2	2.014665
Education and Training	4.393643	1	2.096102
Multidisciplinary Team	7.888416	1	2.808632
**Surgical Wards**			
Age	1.05218	1	1.025758
Male sex	1.037307	2	1.009199
McCabe score	1.294247	3	1.043926
Device number	1.095382	1	1.046605
Hospital Size	34.650296	2	2.426201
Hospital Type	118.763504	3	2.217076
Number of Isolation rooms	64.10419	3	2.000542
IPC-Doctors-to-bed ratio	56.56269	3	1.959243
IPC-Nurses-to-bed ratio	88.931021	3	2.112721
Surveillance of AMR	26.064802	2	2.259507
Surveillance of AMC	34.406337	2	2.421919

Universal Masking	27.311926	2	2.286062
Safety Culture Change	43.759822	2	2.571988
Link with colleagues	11.376844	1	3.372958
**Intensive care units**			
Male sex	1.048747	2	1.01197
Device number	1.132136	1	1.064019
Hospital Location	7.584267	3	1.401691
Number of Isolation rooms	4.091698	3	1.26469
IPC-Doctors-to-bed ratio	10.183553	3	1.472256
IPC-Nurses-to-bed ratio	6.202999	3	1.355502
Surveillance of AMC	4.215365	2	1.432877

Note: AMC, antimicrobial consumption. AMR, antimicrobial resistance. CEO, Chief Executive Officer. Df, degrees of freedom. GVIF, generalised variance inflation factor. IPC, infection prevention and control. MM, multimodal.
