# Supplementary figures and images for: Hospital Context Determinants of Variability in Healthcare-Associated Infection Prevalence: Multi-Level Analysis
Source: Microorganisms. 2024 Dec 7;12(12):2522. doi: 10.3390/microorganisms12122522 (PMC11676765; doi:10.3390/microorganisms12122522)

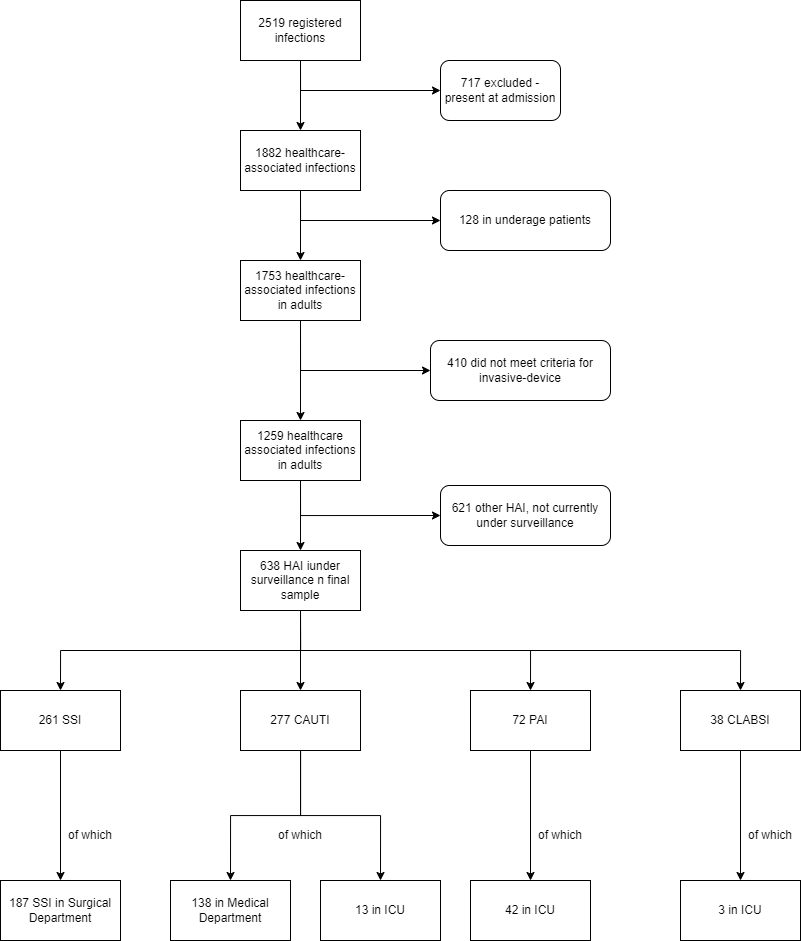

Supplement: Supplementary file 1 [file microorganisms-12-02522-s001.zip › Supp Fig S1.png]
